# Land use efficiency of functional urban areas: Global pattern and evolution of development trajectories

**DOI:** 10.1016/j.habitatint.2022.102543

**Published:** 2022-05

**Authors:** Marcello Schiavina, Michele Melchiorri, Sergio Freire, Pietro Florio, Daniele Ehrlich, Pierpaolo Tommasi, Martino Pesaresi, Thomas Kemper

**Affiliations:** a European Commission, Joint Research Centre (JRC), via E. Fermi 2749, I-21027, Ispra, (VA), Italy; b Fincons Group, Via Torri Bianche, 10, I-20871, Vimercate, (MB), Italy

**Keywords:** SDG 11.3.1, GHSL, Land consumption, Urbanisation, Metropolitan areas, AAPDEA, Abstract Achieved Density in Expansion Area, AOI, Area of interest, BpC, Built-up area per capita, EC, European Commission, FAO, Food and Agriculture Organization, FUA, Functional Urban Area, GDP, Gross Domestic Product, GHS-BUILT, GHSL built-up area spatial grid, GHS-FUA, GHSL FUA layer, GHS-POP, GHSL population spatial grid, GHS-SMOD, GHSL settlement classification spatial grid, GIS, Geospatial Information System, GSARS, Global Strategy on Agricultural and Rural Statistics, HIC, High-Income Countries, LCR, Land Consumption Rate, LCRPGR, Land Use Efficiency indicator, LIC, Low Income Countries, LMC, Lower-Middle Income Countries, LN, Natural logarithm, LUE, Land Use Efficiency, OECD, Organisation for Economic Cooperation and Development, PGR, Population Growth Rate, UMC, Upper-Middle Income Countries, UN, United Nations, UNDESA, United Nations, Department of Economic and Social Affairs

## Abstract

The application of last-generation spatial data modelling, integrating Earth Observation, population, economic and other spatially explicit data, enables insights into the sustainability of the global urbanisation processes with unprecedented detail, consistency, and international comparability. In this study, the land use efficiency indicator, as developed in the Sustainable Development Goals, is assessed globally for the first time at the level of Functional Urban Areas (FUAs). Each FUA includes the city and its commuting zone as inferred from statistical modelling of available spatial data. FUAs represent the economic area of influence of each urban centre. Hence, the analysis of land consumption within their boundary has significance in the fields of spatial planning and policy analyses as well as many other research areas. We utilize the boundaries of more than 9,000 FUAs to estimate the land use efficiency between 1990 and 2015, by using population and built-up area data extracted from the Global Human Settlement Layer. This analysis shows how, in the observed period, FUAs in low-income countries of the Global South evolved with rates of population growth surpassing the ones of land consumption. However, in almost all regions of the globe, more than half of the FUAs improved their land use efficiency in recent years (2000–2015) with respect to the previous decade (1990–2000). Our study concludes that the spatial expansion of urban areas within FUA boundaries is reducing compactness of settlements, and that settlements located within FUAs do not display higher land use efficiency than those outside FUAs.

## Highlights

• Land Use Efficiency (SDG 11.3.1) is assessed in all FUAs of the world.
• FUAs in the Global South show population growing faster than land consumption.
• Development trajectories in FUAs show an improvement of efficiency in recent years.
• Settlements within FUA commuting areas are not more efficient than those outside.
• The proportion of urban centre population does not imply higher efficiency of the FUA.

## 1 Introduction

Urbanisation is one of the megatrends of contemporary human society. This process is traditionally studied by monitoring the demographic and land consumption patterns of an area (Birch & Wachter, 2011). Therefore, the 2030 Development Agenda embedded such aspects in the monitoring framework accompanying the Agenda 2030 for Sustainable Development. The Agenda is based on 17 Sustainable Development Goals (SDGs) that encompass 231 unique indicators and represent a new foundation for the evidence-based monitoring of development policy at global level. Particularly, the SDG 11 (sustainable cities and communities) includes an ambitious target: “*by 2030, enhance inclusive and sustainable urbanization and capacity for participatory, integrated and sustainable human settlement planning and management in all countries*” and proposes a relationship between demographic and land changes to monitor the sustainability of the urbanisation processes with the Land Use Efficiency (LUE) indicator (SDG 11.3.1).

Even if the discussion of the efficiency in land consumption and urbanisation is vibrant (Marquard et al., 2020) in the scientific community, the Sustainable Development Agenda preferred a simplistic but scalable approach to address land use efficiency under a policy standpoint. In contrast, the scientific community considers land use efficiency an interdependent coordination among several disciplines (Liu et al., 2020) and often developed *ad-hoc* metrics to characterize urbanisation according to specific needs (e.g. Hu et al., 2021; Jiao et al., 2020).

The SDG 11 is one of the Goals that requires the integration of Earth Observation (EO) data, to measure settlement extents and characteristics and other data to address urban issues (Siragusa et al., 2020). The pivotal role of the SDG 11 vis-à-vis contemporary societal development, that accounts for the majority of population living in urban areas, and the importance of cities to meet the SDG (Giles-Corti et al., 2020; Klopp & Petretta, 2017), favoured the production of several assessments and studies. In particular, the indicator 11.3.1, “ratio of land consumption rate to population growth rate”, measuring the land use efficiency of a city or an urban area, was computed using a number of land consumption datasets. Studies on this indicator focused on the methods to estimate the land use efficiency in various areas of the globe at multiple scales, from the local one (i.e. single or few cities; Mudau et al., 2020; Philip, 2021), to larger geographical areas (Melchiorri et al., 2019; Nicolau et al., 2018; Schiavina, Melchiorri, et al., 2019). Some examples showcased the use of existing datasets to estimate the indicator, along with a critical review and alternative indicators to complement the original one (Melchiorri et al., 2019; Schiavina, Melchiorri, et al., 2019). In addition to these cases, research institutions have also provided tools to streamline the estimation of the SDG 11.3.1 with *ad hoc* data and for user selected areas of interest (e.g. Trends.Earth^1^ and the Joint Research Centre of the European Commission^2^).

The calculation of the LUE indicator requires first the identification of the spatial entities (i.e. cities or urban areas), in which the land use efficiency will be assessed. The matter of spatial entity definition is a key aspect for LUE as well as for many other SDG indicators, and plays an important role in addressing sustainability issues. The use of a new generation of geospatial data sourced with remote sensing techniques allows a large scale automated identification of settlements that goes beyond administrative designation and supports the study of evolving societal processes. However, the way in which these areas are defined influences indicators and derived conclusions. The methodological guidelines provided by UN-Habitat for the estimation of the SDG 11.3.1 indicate ‘urban areas’ and ‘functional urban areas’ as the spatial entities recommended to conduct such analyses (UN Habitat, 2019). In this manuscript, a first global assessment of the Land Use Efficiency (SDG 11.3.1) indicator is introduced and discussed at the spatial level of the Functional Urban Area (FUA), also named *metropolitan area*. A FUA consists of a city and its commuting zone. Since origin-destination commuting survey statistics are not available globally in a complete form, a statistic inductive model derived from the *Degree of Urbanisation* (Dijkstra et al., 2020) was recently proposed (Moreno-Monroy et al., 2020). This model, based on population density and transportation network data (travelling impedance), combined with economic data, such as the Gross Domestic Product (GDP), produced the spatial baseline dataset (Schiavina, Moreno-Monroy, et al., 2019) used in this study.

Once the area of interest is defined, the computation of the SDG 11.3.1 requires information about population and land consumption. The characteristics of these inputs differ among existing datasets and can influence the indicator estimation. Florczyk et al. (2019) reviewed the semantic heterogeneity among EO derived built-up area products, while Leyk et al. (2019) discussed the characteristics and fitness for purpose of various global population grid products, highlighting advantages and drawbacks of different grids for specific applications such as urban analysis. Shelestov et al. (2020) have recently shown the suitability of the Global Human Settlement Layer (GHSL) datasets for the assessment of land consumption for the SDG Indicator 11.3.1, especially at global level.

The previous analysis conducted by Melchiorri et al. (2019) quantified the SDG 11.3.1, in the period 1990–2015, of 10,000 *urban centres* (dense human settlements of at least 50,000 inhabitants) as defined by the *Degree of Urbanisation*, by using EO derived information produced by the GHSL team. Results indicated that the SDG 11.3.1 has a strong geographic signature and requires additional metrics (like built-up area per capita) for interpretation. The research showed how built-up area per capita varies considerably across *urban centres* with comparable values of LUE. Schiavina, Melchiorri, et al. (2019) expanded this work using the same EO derived information datasets to cover other settlement classes in the urban domain and to estimate their land use efficiency performances. On a regional scale, Jiang et al. (2021) also used GHSL data to assess urbanisation sustainability in China by means of the SDG 11.3.1 indicator, showing improving efficiency trends of urbanisation at country level. With wider territorial focus, still based on the GHSL data, Estoque et al. (2021) evaluated the land use efficiency of all countries at global level. This body of literature suggests that the GHSL data is an established source of information for monitoring the SDG 11.3.1 at different spatio-temporal levels.

However, the territorial scope of the analyses above does not address in details all the designated spatial entities as indicated by the SDG 11.3.1 custodian agency, in particular FUAs. In the context of the land use efficiency, these areas, accounting for 53% of global population in 2015 (Moreno-Monroy et al., 2020), are crucial for the understanding of the impacts of the suburbanisation process that is taking place across the globe, as contemporary urbanisation theory (Brenner & Katsikis, 2020) and evidence suggest (Deng et al., 2021; Gerten et al., 2019; Melchiorri et al., 2018). Therefore, the aim of this study is to analyse the SDG 11.3.1 in all the metropolitan areas (FUA) of the world, leveraging on the global layer produced by Schiavina, Moreno-Monroy, et al. (2019) and on the GHSL spatial grids covering the periods 1990–2000, and 2000–2015, providing insight for policy makers, especially for the identification of focus areas. In these periods, we seek to investigate how land use efficiency and its components have been evolving in and within FUAs, and if this display variations according to country geography and income level.

The next section of the paper presents the material and methods used in the study, and the spatially explicit indicators that are added to characterize the land use efficiency value as per the UNDESA formulation. The results section presents the quantitative results of the experiments by region of the world and by UN 2018 income groups (United Nations, Department of Economic and Social Affairs, Population Division, 2018), then analyses the different performances in the *urban centres* in comparison with the FUA commuting zones. The last section of the results is dedicated to the characterization of the LUE value with built-up area per capita and other spatially explicit metrics. In the discussion and conclusion sections, we highlight some outcomes of our work with respect to existing research on the relationship between land consumption and population growth.

## 2 Material and methods

This research adopts an analytical framework that explores recent empirically-derived geospatial information having a temporal component and global coverage with consistency produced by the GHSL team. This enables the computation of spatially explicit indicators of land use efficiency for FUAs. The methodology is based on the analysis of established and novel metrics characterising land use efficiency in different time periods, in the spirit of the SDG 11.3.1. This approach assesses FUA spatio-temporal patterns and trajectories in this domain, by region of the world and by UN 2018 income groups. The use of detailed and compatible spatial data further enables contrasting the rates of population growth and land consumption, and comparing performance of core versus commuting zones of FUA.

### 2.1 SDG 11.3.1 land use efficiency indicator

Fitting with the research framework outlined above, we start by computing the SDG indicator 11.3.1 (ratio of land consumption rate to population growth rate) using the standard methodology indicated in the UNDESA SDG Metadata Repository.^3^ The custodian agency for this indicator is the United Nations Human Settlement Programme and it classifies the indicator as Tier II (i.e. the “Indicator is conceptually clear, has an internationally established methodology and standards are available, but data are not regularly produced by countries”). The method to compute the indicator instructs to quantify first the rate of land consumption (LCR) and the population growth rate (PGR) in a given spatial unit and time span (*y*). The two rates (LCR and PGR) are computed with Equation (1):

(1) $$LCR=\;\frac{LN\;\left(\frac{Urb_{t+n}}{Urb_{t}}\right)}{y}\;PGR=\;\frac{LN\;\left(\frac{Pop_{t+n}}{Pop_{t}}\right)}{y}$$

In Equation (1), *Urb*_*t*_ and *Urb*_*t + n*_ capture the total areal extent of the land consumed (represented as the extent of the built-up surface of human settlement) at the initial reference year *t* and at the final reference year *t + n*, respectively, while *Pop*_*t*_ and *Pop*_*t + n*_ input the total population of the spatial unit at the initial reference year and at the final reference year, respectively (LN refers to the natural logarithm of the ratio). Equation (2) expresses the calculation of the ratio of land consumption rate to population growth rate that is referred in the UN metadata as LCRPGR, and LUE in this manuscript:

(2) $$LUE\;=\;\frac{LCR}{PGR}$$

LUE_t1_ corresponds to the value calculated in the interval *t1* (in this study 1990–2000), and LUE_t2_ to the one in the interval *t2* (in this study 2000–2015); LUE_dt_ corresponds to the LUE value calculated over the entire period (in this study 1990–2015). As in previous studies and in agreement with UNDESA metadata, LUE values are grouped into five classes aimed at facilitating the interpretation of the indicator. The rationale for this classification is discussed by Schiavina, Melchiorri, et al. (2019) and stands for analysis where LCR is greater than zero (i.e. no decrease of built-up areas in the period). In this scenario, there is only one class representing an efficient development trajectory (0 < LUE ≤ 1) where PGR > LCR, and four classes characterizing inefficient behaviours, with LCR > PGR: LUE < −1; −1 < LUE ≤0; 1 < LUE ≤ 2; and LUE >2.

Given the availability of LUE data for the two time frames (LUE_t1_ and LUE_t2_), we characterized each FUA by improvement or worsening of LUE. Such analysis is performed only for positive values of LUE, being hard to discriminate efficiencies between trajectories generated by negative population growth rates with different LCR paces. Moreover, the nature of the LUE indicator does not allow to compare positive and negative values, an issue well discussed by Schiavina, Melchiorri, et al. (2019). Therefore, limited to cases of population growth (PGR >0 and LUE >0), an improvement of the trend refers to a decrease of the indicator value (LUE_t1_ < LUE_t2_) and a worsening to an increase of the indicator (LUE_t1_ > LUE_t2_). The trends taxonomy includes six cases: three types for worsening and thee for improvement, determined by all the possible combinations of LUE classes for the time intervals *t1* and *t2*.

In addition to the LUE, this research includes two additional indices: one representing the built-up area per capita (BpC_t_ in Equation (3)), and its change between 1990 and 2015 (BpC_dt_ in Equation (4)), plus the Abstract Achieved Population Density in Expansion Areas (AAPDEA in Equation (5)) as introduced by Schiavina, Melchiorri, et al. (2019).

(3) $$BpC_{t}\;=\;\frac{Urb_{t}}{POP_{t}}$$

(4) $$BpC_{dt}\;=\;\left(BpC_{t+n}-BpC_{t}\right)$$

(5) $$AAPDEA\;=\;\frac{Pop_{t+n}-\;Pop_{t}}{Urb_{t+n}-\;Urb_{t}}$$

The spatially explicit metrics in Equations (3), (4), (5) are important tools to characterize the SDG 11.3.1 and help in the LUE interpretation by measuring the urbanisation process from different angles. Newer releases of the UNDESA metadata on this indicator have included considerations concerning the opportunity to add indicators such as the built-up area per capita to characterize the LUE value obtained from Equation (2).

#### 2.1.1 Input data

**Fig. 1:**
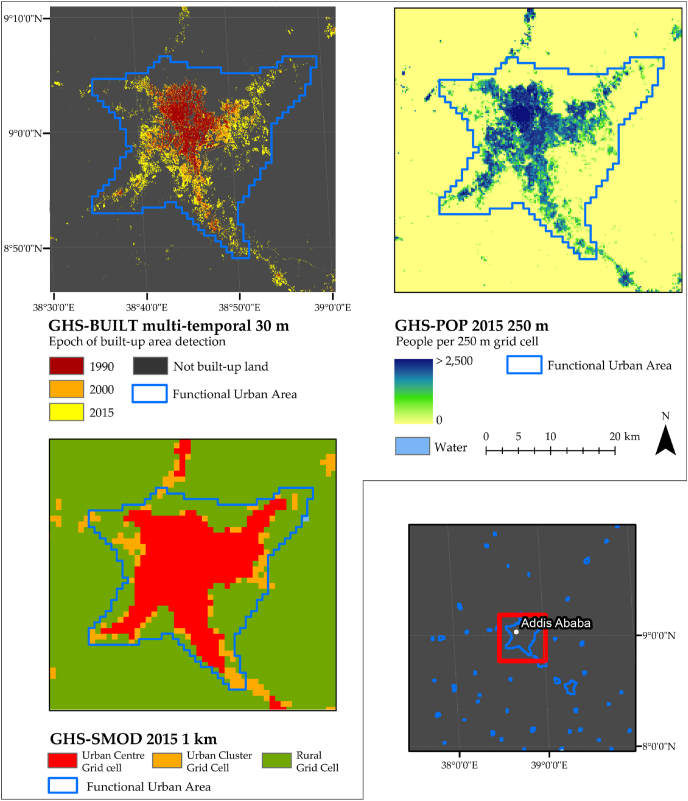
Example of geospatial data used in this study. LCR estimation with GHS-BUILT multi-temporal 1990–2000 -2015 (top left), PGR estimation with GHS-POP multi-temporal (top right) and settlement classification grid GHS-SMOD (bottom left) with FUA delineation in blue in the area of Addis Ababa (Ethiopia). Grading map in the bottom right corner not in scale.

The metadata for the SDG 11.3.1 require information on the delineation of the area of interest, the land consumption and the population growth. This study relies on the following datasets: GHS-FUA for the delineation of the area of interest (AOI) (2.2.2), GHS-BUILT for the LCR information for the epochs 1990–2000–2015 (2.2.3), and the PGR information for the same epochs from GHS-POP (2.2.4). Reporting units are mostly country-level aggregations of FUAs statistics based on the GADM database v2.8^4^ and on the classification of these units by region of the world (UN Major Areas) and income levels as contained in the World Urbanization Prospects 2018 (United Nations, Department of Economic and Social Affairs, Population Division, 2018). Fig. 1 shows an example in Addis Ababa area of the input layers used in this analysis and described in the following sections.

#### 2.1.2 Functional urban area

*Functional Urban Areas* (FUAs) were designated by the United Nations Statistical Commission as one of the extensions of the method recommended for the delineation of urban areas for international statistical comparison (United Nations, 2020). FUAs approximate a metropolitan area by delineating a city along with its commuting zone (i.e. areas from which at least 15% of the resident population commutes toward the main city; OECD, 2012a).

Until recently, delineations of FUAs were only available for OECD countries due to the limited availability of commuting data beyond these. In 2019, Moreno-Monroy et al. (2020) extended the available dataset of FUAs to the global extent by exploiting GHSL datasets (population grid, GHS-POP by Schiavina et al., 2019; and settlement layer, GHS-SMOD by Pesaresi et al., 2019) and other ancillary information (travel time computed from travel impedance, Weiss et al., 2018; and country Gross Domestic Product^5^). By means of a statistical model jointly developed by OECD and JRC, the GHS-FUA layer produced (Schiavina, Moreno-Monroy, et al., 2019) contains the boundary of FUAs for the *urban centres* identified in the GHS-SMOD layer in 2015.

The available vector database (open and free data^6^) incorporates information on 9,032 FUAs including attributes describing the *urban centres* within their boundaries, the country they belong to, their area and the population data in 2015. Population data are available for the whole FUA, for its *urban centres* and for the commuting zone. For the purpose of this study, we expanded the public database by computing the population, for the epochs 1990 and 2000, and the built-up surface, for the epochs 1990, 2000 and 2015, by means of zonal statistics processing in GIS environment and keeping the FUA boundary fixed. Built-up area and population estimates are obtained using the datasets GHS-BUILT and GHS-POP, respectively, presented in the next sections. Due to some anomalies in the built-up area data of past epochs (e.g. missing built-up in the FUA) we have removed from the analysis 13 FUAs that would have been associated to unrealistic LUE values (i.e. infinite).

#### 2.1.3 Land consumption

In this study – to be compatible with previous research on this indicator (Melchiorri et al., 2019; Schiavina, Melchiorri, et al., 2019) - we use the built-up layer produced by GHSL as information source to estimate land consumption (LCR) between 1990 and 2015. This choice, further to the reasons presented in Melchiorri et al. (2019) related to the worldwide coverage, thematic fitness (semantic), temporal coverage (and change analysis suitability), open and free data policy, is also justified by the need to cover all the *urban centres*, plus their surrounding areas, globally.

GHS-BUILT (Corbane et al., 2018) is a global and multi-temporal grid that expresses the density of built-up area obtained by processing large volumes of EO data with a symbolic machine learning process applied to Landsat data collections plus a Sentinel-1 and GlobeLand30 learning sets (Corbane et al., 2019). Table 1 synthetizes the key characteristics (semantic, input, epoch and grid cell resolution) for the GHS-BUILT layer used in this study. In this research, *Urb*_*t*_ and *Urb*_*t + n*_ in Equations (1), (3), (4), (5) are the sum of all built-up areas at the grid level in the GHS-BUILT layers for epochs 1990–2000–2015 (according to the time interval investigated) for the AOI obtained via zonal statistics in GIS environment.

**Table 1:** Comprehensive overview of the dataset used in the study with semantic, baseline data, epoch and grid cell resolution.

Name	Semantic	Main Input Data	Epoch	Grid Resolution
**GHS-BUILT**	Density of built-up area per grid cell	Satellite imagery (Landsat collections and Sentinel-1)	1990 2000 2015	1 km
**GHS-POP**	Resident population counts per grid cell	Census data (CIESIN GPW v4.10), GHS-BUILT		
**GHS-SMOD**	Settlement classification (urban centres, urban clusters, and rural grid cells)	GHS-BUILT, GHS-POP		
**GHS-FUA**	Functional urban area of urban centres	GHS-BUILT, GHS-POP, travel time, country GDP	2015	

#### 2.1.4 Population growth

Population growth is quantified, similarly to previous studies (Melchiorri et al., 2019; Schiavina, Melchiorri, et al., 2019), by computing the growth rate of population (PGR) derived from the population grids produced by GHSL. As for the GHS-BUILT, the global geographical coverage, the temporal coverage, the semantic (resident population), the open and free data policy, and also the alignment with GHS-BUILT grids, motivated this selection.

The GHS-POP (Schiavina et al., 2019) is a global and multi-temporal raster dataset that represents the abundance of population obtained by downscaling the harmonized estimates of resident population produced by the Center for International Earth Science Information Network (CIESIN) in their GPW project (Center For International Earth Science Information Network-CIESIN-Columbia University, 2017) from globally collected census data. The characteristics of this dataset as used in this study are reported in Table 1. In this research, *Pop*_*t*_ and *Pop*_*t + n*_ in Equations (1), (3), (4), (5) are the sum of the population, at the grid level, in the GHS-POP layers for the epochs 1990–2000–2015 (according to the time interval analysed) for the areas of interest obtained via zonal statistics in GIS environment.

## 3 Results

This section presents the results mainly by geographical region of the world (according to UNDESA grouping by Major Area), and by income level. These groupings returned specific SDG 11.3.1 patterns. The statistical dataset including land use efficiency values for all FUAs is provided in supplementary material S1.

### 3.1 The land use efficiency in the global south and in areas where the gross national income per capita is below global average shows more efficient development trajectories

Between 1990 and 2015, FUAs expanded their built-up area by almost 130 × 10^3^ km^2^ (48% of the global built-up areas expansion) and added 1.13 × 10^9^ people globally (56% of the global population growth). This corresponds to a LUE_dt_ value of 1.01. However, the relationship between spatial expansion and demographic growth is very diverse across regions of the world (Table 2). For example, FUAs in Asia are responsible for about 44% of the land consumption in all FUAs worldwide and 58% of the population growth, while Africa accounts for about 20% of global population growth in global FUAs, but only 6% of the built-up area expansion in these areas. Europe contributed with 3% of the global FUA demographic change, and 13% of spatial growth of built-up, while North America with 5% and 29%, respectively. LUE_dt_ is considerably below 1 in Africa (0.5) and Oceania (0.6), and almost equal to 1 in Latin America & Caribbean (0.9): these cases correspond to a population growth rate higher than that of the land consumption (PGR > LCR). LUE_dt_ is above 1 in Europe (2.9), Northern America (1.5), and Asia (1.2) (LCR > PGR). By splitting the time interval in two sub-periods (LUE_t1_ 1990–2000 and LUE_t2_ 2000–2015) all regions of the world show an improvement of LUE value in the most recent period.

**Table 2:** Land Use Efficiency values for different geographic and income grouping of countries for the respective epochs; *t1* refers to the period 1990–2000, *t2* to the period 2000–2015 and dt to the period 1990–2015.

Grouping	LUE_t1_	LUE_t2_	LUE_dt_
Africa	0.9	0.3	0.5
Asia	1.5	0.9	1.2
Europe	4.3	2.0	2.9
Latin America & Caribb.	1.3	0.6	0.9
Northern America	2.2	0.9	1.5
Oceania	0.9	0.4	0.6
High-income	2.3	1.0	1.5
Upper-middle income	1.7	1.1	1.4
Lower-middle income	1.4	0.6	0.9
Low-income	0.8	0.3	0.5
**World**	**1.4**	**0.7**	**1.0**

The classification of countries by income levels also shows a clear pattern of decreasing efficiency in development trajectories with increasing income. Namely, in High-Income Countries (HIC), land consumption and population growth rate are in a relationship ratio of 1.5 (LCR > PGR) between 1990 and 2015. Upper-Middle Income Countries (UMC) record a similar value (LUE_dt_ = 1.4). The relationship between land consumption and population growth rate changes in Lower-Middle Income Countries (LMC) with PGR greater than LCR (LUE_dt_ = 0.9). An even greater dominance of PGR over LCR is tracked in Low-Income Countries (LIC) (LUE_dt_ = 0.5). Also, in the case of grouping by income, the LUE values in the period 2000–2015 for all levels show a more efficient behaviour than the one in the period 1990–2000.

**Fig. 2:**
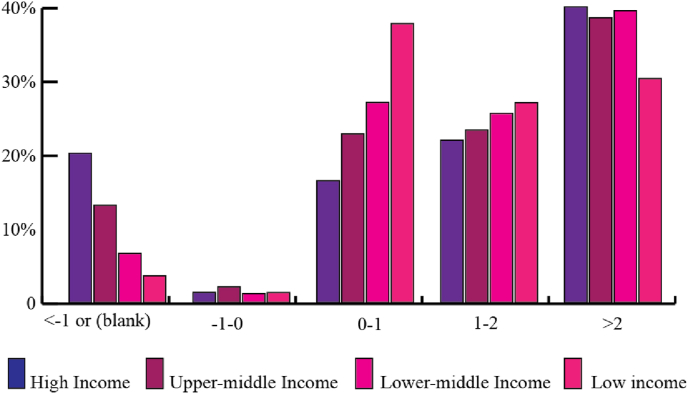
Frequency distribution of FUAs by LUE class and income class.

Another trait of the LUE in the FUAs is the frequency distribution across the income levels (Fig. 2). In particular, 38% of the FUAs in LIC and 27% in LMC show a LUE trajectory with PGR > LCR. By contrast, less than 25% of the FUAs in HIC and UMC belong to this class. High-Income Countries distribution peaks in the LUE >2 class with 40% of the FUAs in the class; moreover, 20% of the FUAs in HIC decline in population and expand in built-up land (LUE < −1). UMC have characteristics similar to HIC (higher frequency in classes LUE >2 and LUE < −1) and LMC to LIC (higher frequency in class 1 < LUE ≤ 2).

#### 3.1.1 Geographical distribution of FUAs by LUE class

**Fig. 3:**
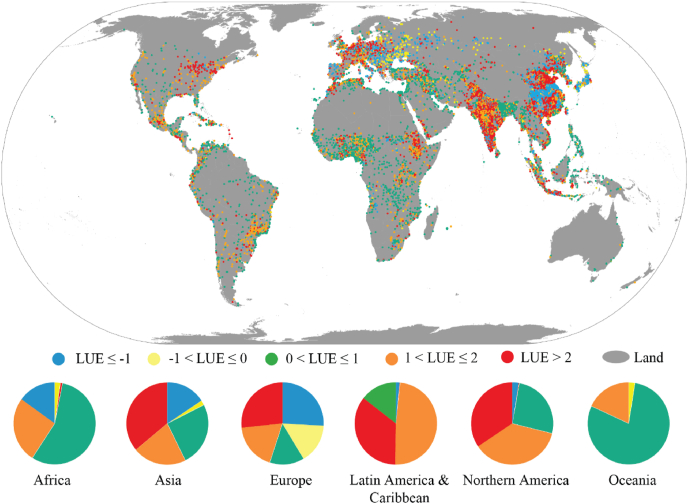
Global map of Land Use Efficiency indicator in functional urban areas estimated with GHSL data between 1990 and 2015.

LUE has been estimated for 9,019 FUAs, and Fig. 3 displays a comprehensive overview of the SDG 11.3.1 quantified with the GHSL data between 1990 and 2015 (LUE_dt_). Overall, in 26% of the FUAs of the world (2,974), the LUE_dt_ value is between 0 and 1 (PGR > LCR), represented as green in Fig. 3. FUAs in this class are mainly located in Asia and Africa (43% and 34%), Latin America (15%), with few of them being in Europe and Northern America (8% and 5% respectively). The vast majority of the 1,099 FUAs with LUE < −1 is concentrated in Asia and Europe (73% and 21%, respectively). A similar distribution is tracked for the class −1 < LUE ≤0 where Europe and Asia host 57% and 36% of the FUAs in this class (245 FUAs). These latter two LUE classes capture FUAs that suffered demographic decline (PGR <0) and built-up land expansion (LCR >0). Half of the FUAs in the LUE class 1 < LUE ≤ 2 (2,134 FUAs) are located in Asia, 22% in Africa, 15% in Latin America & Caribbean. The 2,568 FUAs in the class LUE >2 are mostly in Asia (71%), with about 10% in Africa and Europe.

The charts in Fig. 3 show that the absolute majority of the FUAs (56%) in Africa are in the class 0 < LUE ≤ 1, and 26% in the class 1 < LUE ≤ 2. A relative majority of the FUAs in Asia (36%) expand in built-up areas at a rate at least double the one of population growth (LUE > 2), while 25% of the FUAs have an efficient trajectory of PGR > LCR (0 < LUE ≤ 1), and 21% of the FUAs in this region are in the class 1 < LUE ≤ 2. In Europe, 41% of the FUAs expand in the built-up areas but decline in population (aggregation of LUE < 0 classes), 27% of the FUAs expand in built-up areas at a rate at least double the one of population growth, while only 14% of the FUAs have a LCR < PGR. In Latin America & Caribbean almost half (49%) of the FUAs have an efficient trajectory of LCR < PGR; in 35% of the FUAs and another 14% LCR prevails over PGR with a rate up to a factor of 2 and exceeding 2, respectively. FUAs in Oceania are mostly in the class 0 < LUE ≤ 1 (79%), and 18% in the class 1 < LUE ≤ 2, the remaining 3% is in the class LUE < −1.

#### 3.1.2 LUE trends between 1990-2000 and 2000–2015 periods

**Fig. 4:**
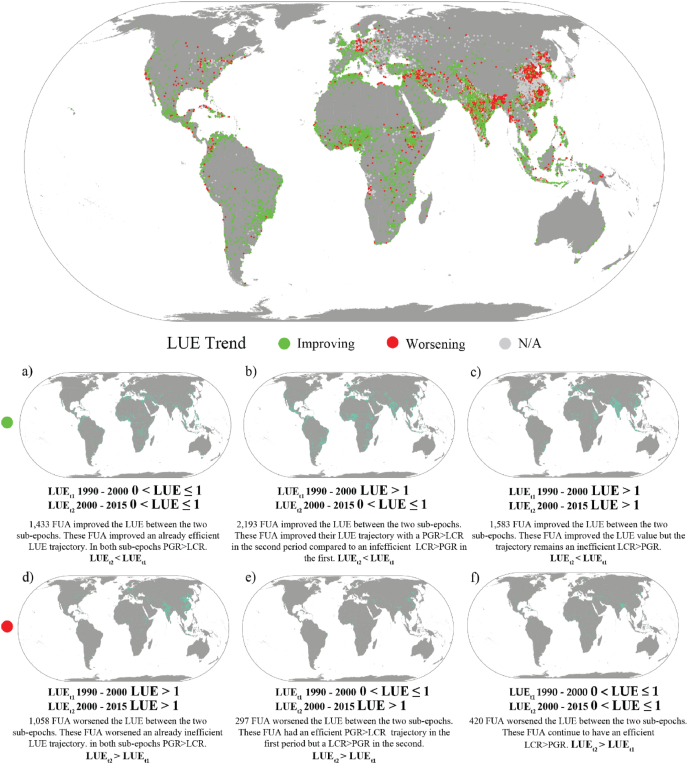
Global map of the Land Use Efficiency indicator trend in functional urban areas, estimated with GHSL data between 1990– 2000 and 2000–2015. N/A records correspond to FUAs with a LUE <0 in LUE_t1_ or LUE_t2_. Each box shows the distribution of FUAs according to their LUE trend.

The observation of LUE_t1_ and LUE_t2_ was implemented to identify reversal, continuation and other trends of LUE in FUAs of the world. Fig. 4 shows the LUE trends in the 9,019 FUAs in the database of this study.

Table 3 reports the frequency of the changes in LUE value between 1990-2000 and 2000–2015 periods. In 58% of the FUAs (5,209) the LUE improved in 2000–2015 compared to 1990–2000, while in 20% (1,775 FUAs) it worsened. The lower section of Fig. 4 characterizes and locates the FUAs according to the LUE trend determinants.

**Table 3:** Frequency distribution of changes classes in LUE values between the 1990–2000 and 2000–2015 periods.

Class	LUE_t1_	LUE_t2_	Frequency
*Improving LUE(LUE*_*t2*_ *<* *LUE*_*t1*_*)*			
a)	0 < LUE_t1_ < 1	0 < LUE_t2_ < 1	1,433
b)	LUE_t1_ > 1	0 < LUE_t2_ < 1	2,193
c)	LUE_t1_ > 1	LUE_t2_ > 1	1,583
*Worsening LUE(LUE*_*t2*_ *>* *LUE*_*t1*_*)*			
d)	LUE_t1_ > 1	LUE_t2_ > 1	1,058
e)	0 < LUE_t1_ < 1	LUE_t2_ > 1	297
f)	0 < LUE_t1_ < 1	0 < LUE_t2_ < 1	420

The absolute majority (60%) of the FUAs that worsened their LUE between *t1* and *t2* are in the non-efficient and worsening type (Fig. 4d) due to a LUE_t1_ > 1 and LUE_t2_ > 1 and LUE_t2_ > LUE_t1_. More than 15% of the FUAs in Asia and 13% of the ones in Northern America fall in this category. FUAs in this class include: Osaka and Fukuoka (JPN), Suzhou, Shenyang and Qingdao (CHN), Berlin and Cologne (DEU), Boston, St. Louis, and Baltimore (USA). About 15% of the FUAs with a worsening trend significantly reversed their development pattern, moving from an efficient LUE_t1_ trajectory (0 < LUE ≤ 1) to an inefficient one in LUE_t2_ (LUE > 1). This class is the least frequent across regions of the world with a distribution peaking at 6% in Northern America, while in other regions this type representativeness is below 5% (Fig. 4e). Examples of FUAs in this class include: Hefei and Nanchang (CHN), Frankfurt and Stuttgart (DEU), and Darjeeling (IND).

The last typology of worsening trajectory, with about 25% of the worsening FUAs, is the one of LUE_t1_ < LUE_t2_ with 0 ≤ LUE ≤ 1 in both time intervals. This class captures those FUAs that were and remain in a PGR > LCR relationship but the ratio worsens. About 10% of the FUAs in Oceania, and 5% of those in Northern America and Africa are in this class (Fig. 4f). Examples of FUAs in this class include: Taipei and Tianjin (CHN), Colombo (LKA), Kano (NGA), Kabul (AFG), Santo Domingo (DOM), and Dakar (SEN).

Of the 5,029 FUAs that improved LUE, 42% significantly reversed their development pattern moving from an inefficient LUE_t1_ trajectory (LUE > 1) to an efficient LUE_t2_ (0 < LUE ≤ 1). About 20% of the FUAs in Northern America, Europe and Asia are in this category (Fig. 4b). Examples of FUAs in this class include 11 FUAs with more than 11 million inhabitants in 2015, such as Tokyo (JPN), Delhi and Chennai (IND), Cairo (EGY), Guangzhou (CHN), Moscow (RUS), Los Angeles (USA), Buenos Aires (ARG), Lahore (PAK), and Paris (FRA), plus other 92 exceeding 2 million inhabitants in 2015, like Kinshasa (COD), Addis Ababa (ETH), Rome (ITA), Kumasi (GHA) and Jakarta (IDN).

Improvements took also place in 1,583 FUAs despite having inefficient trajectories (LUE_t1_ > 1 and LUE_t2_ > 1) but LUE_t2_ < LUE_t1_. Almost half of the FUAs follow this trajectory in Latin America and the Caribbean, about 40% of those in Africa, and about 30% of those in Northern America and Oceania (Fig. 4c). The last typology of improvement identifies FUAs (28% of improving efficiency) that had an already efficient PGR > LCR relationship (0 < LUE ≤ 1) in *t1*, and improved it in *t2* (LUE_t2_ < LUE_t1_). More than 40% of the FUAs in Oceania, 30% of the FUAs in Africa and 25% of the FUAs in Latin America and the Caribbean are in this group (Fig. 4a). This class includes some of the very large FUAs (more than 10 million inhabitants in 2015) like Seoul (KOR), Mexico City (MEX), New York (USA), and more than 90 FUAs exceeding 2 million inhabitants (in 2015) including: Wuhan and Ningbo (CHN), Chicago and Philadelphia (USA), Kyiv (UKR), Rotterdam (NLD), Warsaw (POL), Budapest (HUN), Lisbon (PRT).

This worsening/improving analysis is not available for 2034 FUAs, 23% of all FUAs in the world (grey markers in Fig. 4) where at least one value of LUE in *t1* or *t2* is negative. These are almost 50% of the FUAs in Europe, 27% of the FUAs in Asia and 20% of the FUAs in Northern America.

### 3.2 Demographic and built-up area change profiles are substantially different within FUAs: urban centres generally densify while commuting zone density decreases

Functional urban areas are entities composed by the *urban centre* plus the commuting zone. Commuting zones include different settlement typologies (by *Degree of Urbanisation*). Worldwide, *urban centre* grid cells account for 1/4 of the FUA area (627,301 km^2^), 1/5 of the area is *urban cluster* (531,052 km^2^), with the remaining 54% (1,378,333 km^2^) being *rural areas*.

Previous research highlighted how, in *urban centres,* the relationship between LCR and PGR is in general PGR > LCR (Melchiorri et al., 2019), and that the *Degree of Urbanisation* settlement classes other than *urban centre* have a LCR > PGR relation (Schiavina, Melchiorri, et al., 2019).

#### 3.2.1 LUE in FUAs 1990–2015: urban centres vs commuting zone

**Fig. 5:**
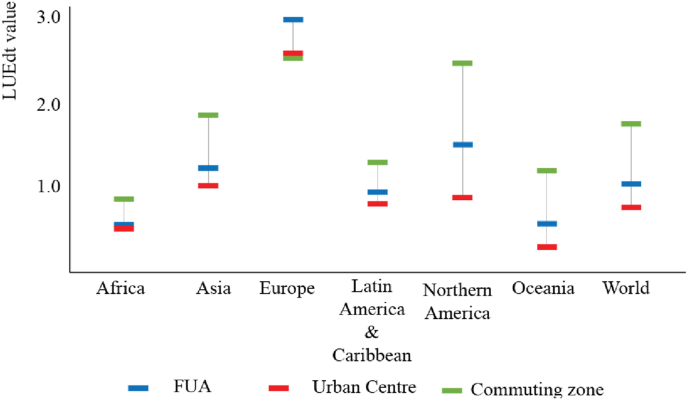
Comparison of LUE in FUA (blue), urban centre (red) and commuting zones (green) by region of the world between 1990 and 2015.

In the period 1990–2015, *urban centres* confirm a lower value of LUE (LUE_dt_ = 0.75) compared to commuting zones (LUE_dt_ = 1.69) (Fig. 5). Values for commuting zones vary across the globe in a way similar to the variability of LUE in *urban centres* analysed in Schiavina, Melchiorri, et al. (2019). Between 1990 and 2015, commuting zones added 175.5 × 10^6^ people (+36.2%) and built-up areas expanded by about 63.7 × 10^3^ km^2^ (+68.7%). In Europe and Northern America, the LUE relative to commuting zones (LUE_dt_) is greater than 2. Commuting zones in Asia, Latin America and the Caribbean, and Oceania also have a LCR > PGR relationship but the LUE value is generally lower (1 < LUE ≤ 2). Only commuting zones in Africa have LCR < PGR (0 < LUE ≤ 1). Europe is the only region where the LUE_dt_ value of the commuting zones is lower (2.42) than the one of the *urban centres* (2.49).

### 3.3 Spatially explicit indicators to characterize LUE in FUAs

#### 3.3.1 Built-up area per capita in FUAs

Built-up area per capita (BpC) is one of the spatially explicit indicators that can assist in further characterizing LUE values. In FUAs of the globe, BpC declined by 8.2 m^2^ per person between 1990 and 2015 to 89.1 m^2^ per person (Table 4). Initial and final BpC vary across regions in absolute terms, but the only region where BpC has increased is Europe (+10.1 m^2^ per person). Between 1990 and 2000, BpC increased in Asia (+3.2 m^2^ per person), Latin America & the Caribbean (+3.0 m^2^ per person) and Northern America (+12.8 m^2^ per person), but it decreased between 2000 and 2015 (−3.3, −9.5 and −27.4 m^2^ per person, respectively). Although the LUE in Asia and Latin America and Caribbean nominally worsened in 1990–2000, they departed from low BpC, and their BpC remained below the global average at the end of the period. In 2015, BpC in the African FUAs remains a fraction of that in Northern America (1/10) and Europe (1/4), and over time, BpC in Africa has even declined (−17.6 m^2^ per person). BpC also varies within FUAs: in the *urban centres* is on average 0.9 times the value in the FUAs, while BpC in commuting zones is on average 1.5 times the one in the FUAs.

**Table 4:** Built-up area per capita (BpC) and BpC change (BpC_dt_) in m^2^ per person in 1990–2000 – 2015 in Functional Urban Areas, by region of the world.

Region	BpC (m^2^ per person)			BpC_dt_ (m^2^ per person)		
	1990	2000	2015	1990–2000	2000–2015	1990–2015
Africa	64.7	62.4	47.1	−2.3	−15.3	−17.6
Asia	61.9	65.1	61.8	3.2	−3.3	−0.1
Europe	148.0	156.1	158.0	8.2	1.9	10.1
Latin America & Caribbean	89.0	92.0	82.5	3.0	−9.5	−6.5
North America	414.7	427.5	400.1	12.8	−27.4	−14.6
Oceania	368.4	347.9	288.9	−20.5	−59.0	−79.5
**World**	**97.3**	**98.6**	**89.1**	**1.3**	**−9.5**	**−8.2**

#### 3.3.2 Effects of FUA population size on LUE

**Fig. 6:**
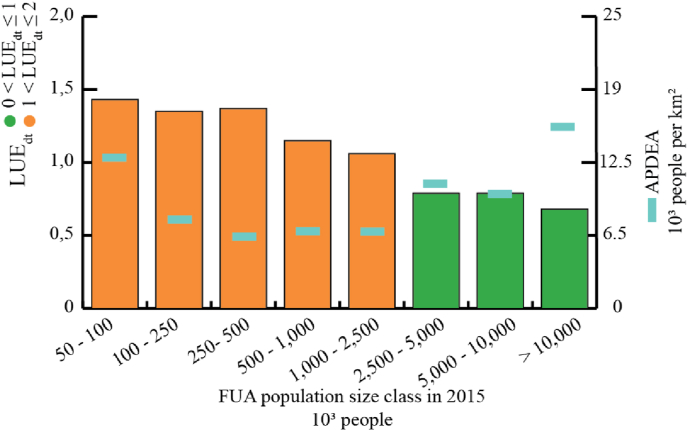
LUE values (left vertical axes) in FUAs by population size class (2015) compared with AAPDEA (right vertical axes); bars are colour coded by LUE value range.

The Abstract Achieved Population Density in Expansion Areas (AAPDEA) identifies the relationship between new population and the new built-up areas within the FUA (see Equation (5)). As for the BpC, this spatially explicit metric helps analysing (or characterizing) the LUE value. Fig. 6 shows the breakdown variation of the AAPDEA (new inhabitants per km^2^ of new built-up area) between 1990 and 2015 in FUAs of the world, by population size class. The chart shows that despite larger FUAs displaying lower LUE values, AAPDEA varies and declines until FUAs of 500,000 inhabitants and then tends to increase. This indicator shows that although FUAs in the population size class 50,000–100,000 people (small size FUAs) have a LUE_dt_ > 1, one square kilometre of built-up areas expansion relates to almost 13,000 new inhabitants, and a BpC in 2015 equivalent to 58 m^2^ per person. In larger FUAs (i.e. from the class 2.5-5 million people), LUE identifies efficient development trajectories (0 < LUE_dt_ < 1) and each square kilometre of new built-up areas expansion relates to more than 10,000 new people and the BpC in 2015 below 130 m^2^ per person.

## 4 Discussion

The relationship between expansion of built-up areas and population dynamics is a traditional driver of urbanisation (Angel et al., 2011). The SDG 11.3.1 leverages on such relation, but an increasing body of knowledge documents divergence from a linear or causal relationship between land consumption rate and population growth rate. Early studies by Seto et al. (2011, 2012) already identified an expansion of global urban areas at a pace twice as fast as their population growth.

The results of our analysis confirm the findings of Estoque et al. (2021), Jiang et al. (2021) and Seto et al. (2011, 2012), and identify other specific characteristics of land use efficiency, calculated in accordance to the SDG metadata. Furthermore, when dissecting the LUE value with the additional spatially explicit indicators computed in this study, different findings emerge.

### 4.1 Improvement or worsening of LUE shows no evident relationship with FUA population size, but strong regional signature and ties with suburbanisation processes

Our analysis identifies that development trajectories in FUAs are getting more sustainable, with about 40% of all FUAs of the world that developed with an efficient dynamic between 2000 and 2015, improving their LUE assessed in the previous decade (1990–2000). Only 15% of the FUAs worsen their trajectory leaving an efficient path (3%) or worsening their inefficient development (12%). Declining urban centre population and growing population in commuting areas characterize 21% of these FUAs experiencing a worsening of LUE value. These population dynamics are much less frequent in FUAs where the LUE is improving (only 1%). Therefore, these patterns may potentially denote an intensification or the onset of suburbanisation processes.

We inspected the possibility of a relationship between these trends and the size of each FUA. However, despite the results presented in section 3.3.2 showing that LUE decreases when FUA population size increases (in line with the theoretical hypothesis of a higher efficiency of larger agglomerations; He et al., 2020), the population size of the agglomeration has no effect when it comes to LUE trends over time (see supplementary material S2).

Continued expansion in built-up areas and low population growth rates, especially in *urban centres*, are frequent across Europe. This unique feature makes Europe the only region with a persistent increase in built-up area per capita. Moreover, the low or even negative population growth rates of European *urban centres* imply a lower efficiency of these settlements with respect to the commuting areas – a phenomenon only observed in Europe. This peculiar behaviour requires specific in-depth analyses of the European territory in order to better understand these dynamics and develop specific policies addressing population decline.

### 4.2 The proportion of population located in the urban centre (the core of the FUA) does not correlate with a higher land use efficiency of the FUA

LUE research in *urban centres* shows that this settlement type has in general a high level of land use efficiency as calculated with UNDESA SDG 11.3.1 method (Melchiorri et al., 2019). When analysing LUE in FUAs, it is important to determine if the proportion of population in the *urban centre* with respect to the FUA population has an effect on the LUE of the FUA. When grouping FUAs according to the proportion of FUA population in the *urban centre*s, no clear relationship with the LUE emerges (see supplementary material S3). The LUE in FUA is around 1 irrespectively of the proportion of the FUA population in the *urban centres*, except when this proportion is below 0.2 (LUE = 2).

### 4.3 Settlements belonging to FUAs are not more efficient compared to settlements outside FUAs

Previous research on LUE identified higher land use efficiency in *urban centres* compared to other settlement classes identified by the *Degree of Urbanisation* (*urban centres* LUE = 0.72; *urban clusters* LUE = 1.57; *rural areas* LUE = 1.99; Schiavina, Melchiorri, et al., 2019). Our study compared the LUE calculated in commuting zones of FUAs to the one of settlements outside FUAs and found that settlements within FUAs have a higher LUE_dt_ value (1.69) compared to LUE_dt_ of settlements outside FUAs (1.27). Between 1990 and 2015, built-up areas in commuting zones increased by 68% compared to 50% outside FUAs of the globe. In the same period, population increased by 36% in commuting zones and by 38% in settlements outside FUAs. This finding is particularly relevant for territorial planning policy, because it may imply that settlements expansion close to urban hotspots (the *urban centres*) are less efficient than settlements elsewhere (which could even be largely rural ones).

### 4.4 Spatial expansion of urban areas within FUAs is reducing compactness of settlements with patterns not related to any other indicator (LUE, LCR, GDP)

An overall decrease in compactness of *urban clusters* within FUAs is observed in the time period 1990–2015, reflecting a inefficient expansion of urban areas from a morphological perspective, possibly due to physiographic constraints (see supplementary material S4). This trend is particularly pronounced in areas like Eastern Africa, Eastern China and Northern India: regardless of cities expansion policy, a decreasing compactness can be harmful for climate change mitigation measures and several environmental aspects. Less compact human settlements consume more resources (OECD, 2012b), put protected areas under pressure (Fuente et al., 2020), increase concentrations of air pollutant (Crippa et al., 2021), threaten biodiversity and affect ecosystem productivity (Seto et al., 2012). There seems to be no correlation between compactness and other variables (i.e. LUE, LCR, FUA area, income), not even in larger FUAs (>500K inhabitants), which is consistent with recent literature (Angel et al., 2020), conducting similar research on other datasets (see supplementary material S4 for details).

The results presented in this study align with other findings on research applied to the SDG 11.3.1 (Cai et al., 2020; Kussul et al., 2020; Nicolau et al., 2018), which identified an overall relationship LCR > PGR for settlements around the globe, irrespectively of spatial units of analysis. Possible sources of uncertainty may derive from data scarcity for the delineation of functional urban areas and their relative attributes (multi-temporal built-up area and population) for certain areas of the globe, as described by Moreno-Monroy et al. (2020). Land consumption rate may be affected by limitations in the built-up areas detection workflow (relying on physical observability of built-up area structure) as presented by Corbane et al. (2019). Moreover, the land component is quantified by changes of built-up surfaces detected from Landsat imagery, while in recent epochs, larger and wealthier cities tend to have significant transformations in the vertical component of buildings (i.e. taller). The AAPDEA is not able to control for floor area density, and therefore, in such conditions, it can be biased towards higher densities of new inhabitants per unit of new built-up land. Pesaresi et al. (2021) demonstrate the capability to produce generalized estimates of built-up volume required to quantify urban indicators in high demand (Angel et al., 2021).

Population estimates may also contribute to uncertainty due to limitations in source data and harmonisation procedures (Freire et al., 2018). Besides these sources of uncertainty, the research demonstrates the pivotal role of EO to support SDG estimation with a key contribution to establish databases for monitoring of indicators. In this respect, the SDG 11.3.1 requires the integration of EO data with other socio-economic data. This requirement urges the remote sensing community to widen its spectrum of applications, embracing multidisciplinary cooperation with other sectors and expertise.

## 5 Conclusions

The estimation of the SDG 11.3.1 (ratio of land consumption rate to population growth rate, namely Land Use Efficiency) aims at tracking the relationship between spatial expansion and population change of settlements in urban areas. Earth Observation is a key source for measuring land consumption rate because the delineation, characterization and monitoring of human settlements over time are core thematic domains in the EO community. This research presents a concrete example of EO support for SDG applications. Moreover, it demonstrates that the estimation of the SDG 11.3.1 is possible with open data and at global scale. Furthermore, as the LUE does not consider a baseline regarding population density and Built-up per capita (thus avoiding any autocorrelation with the FUA boundary estimation), the analysis is complemented with additional metrics.

The specific formulation of the indicator requires synergies and integration of EO information with data coming from other disciplines (demography and urban studies). In this research, we presented the estimation of the SDG 11.3.1 in 9,019 Functional Urban Areas of the globe between 1990 and 2015. FUAs are one of the extensions of the *Degree of Urbanisation*, the method endorsed by the United Nations Statistical Commission United Nations (2020) to delineate urban areas for international comparison, and are a suitable spatial extent for the indicator calculation suggested by the custodian agency. Land use efficiency has been estimated with the Global Human Settlement Layer (GHSL) data by computing the land consumption (with the GHS-BUILT layer) and the population growth rate (with the GHS-POP layer) within the delineated areas of interest (with the GHS-FUA layer). The GHSL proved suitable for our analysis, which required population, built-up area and settlement information for the entire globe and over multiple time steps (1990–2000–2015). Beside this capabilities and high thematic accuracy of the input data, better information collected and processed in the future (Sentinel-2 imagery and geocoded census) will further improve the precision of the output. Further research is needed on the sensitivity of the LUE indicator to different typologies of input data regarding the LCR component. In this regard, several products are available to date, yet few retain full multi-temporal suitability or are available as open and free data.

Current research on land change dynamics due to human settlement expansion all concluded that at global level there is a general trend of a continued natural resource depletion. In particular, many researchers highlighted how the rate of land consumption outpaced the population growth rate. Our research concludes that overall, between 1990 and 2015, this trend is confirmed in commuting zones. However, considering all the functional urban areas of the globe, the rate of land consumption equals the population growth rate, with a significant improvement of efficiency in recent years. Demographic and built-up area changes are substantially different within FUA settlements. *Urban centres* generally densify (i.e. increasing efficiency), while the density of commuting zones decreases (i.e. reducing efficiency), reflecting the general scaling theory of higher efficiency of larger agglomerations. Nevertheless, this general result is subject to several variations in space. As detected in previous research focusing on different spatial entities and extent, the economic affluence and the geographic location greatly affect the efficiency of settlement land use. Land use efficiency value is lower (i.e. more efficient) in the Global South and in areas where the gross national income per capita is below global average.

These efficiency estimations in the urban rural continuum are especially relevant for spatial and territorial planning to address development control. Urban expansion pressure can lead to a divergence between built-up and population growth trajectories and therefore to inefficient LUE trends. Policy makers might greatly benefit from the results of this analysis that could help in the identification of focus areas (e.g. the commuting zones) where to concentrate efforts and possibly learn from efficient cases.

Generally, we can conclude that there is an overall improvement of land use efficiency in recent years. To confirm this trend and carry out a continuous monitoring of the SDG 11.3.1 as requested in the Sustainable Development Agenda, periodic updates of the GHS-BUILT database and GHSL products under the Copernicus programme of the European Union would be a strong asset.

## Author contributions

Conceptualization, M.M. and M.S.; methodology, M.S, M.M. and S.F.; software, M.S; validation, M.S; formal analysis, M.M, M.S. and P.F.; data curation, M.S; writing—original draft preparation, M.M.; writing—review and editing, M.S, M.M., S.F., P.F., P.T., M.P., D.E. and T.K.; visualization, M.M.; supervision, M.S. and M.P.; project administration, T.K. All authors have read and agreed to the published version of the manuscript.

## Data availability statement

Input data for this study (GHSL baseline data) can be freely downloaded from: https://ghsl.jrc.ec.europa.eu/download.php.

## Disclaimer

The designations employed and the presentation of materials and maps do not imply the expression of any opinion whatsoever on the part of the European Union concerning the legal status of any country, territory or area or of its authorities, or concerning the delimitation of its frontiers or boundaries that if shown on the maps are only indicative. The boundaries and names shown on maps do not imply official endorsement or acceptance by the European Union. The views expressed herein are those of the authors and do not necessarily reflect the views of the European Union.

## Declaration of competing interest

Authors declare no conflict of interest.

## Footnotes

1 http://Trends.Earth/Docs/En/Background/Understanding_indicators11.Html (Accessed on 28/05/2021).

2 https://ghsl.jrc.ec.europa.eu/tools.php (Accessed on 28/05/2021).

3 https://unstats.un.org/sdgs/metadata.

4 https://gadm.org/.

5 https://data.worldbank.org/indicator/NY.GDP.PCAP.CD.

6 https://ghsl.jrc.ec.europa.eu/download.php.
