# Supplementary material for: Land use efficiency of functional urban areas: Global pattern and evolution of development trajectories
Source: Habitat Int. 2022 May;123:None. doi: 10.1016/j.habitatint.2022.102543 (PMC9097785; doi:10.1016/j.habitatint.2022.102543)
Supplement: Multimedia component 2 [file mmc2.docx]

**Global estimation of SDG 11.3.1 in functional urban areas**

Marcello Schiavina, Michele Melchiorri, Sergio Freire, Pietro Florio, Daniele Ehrlich, Pierpaolo Tommasi, Martino Pesaresi, and Thomas Kemper

**Supplementary material (S2, S3 and S4)**

S2


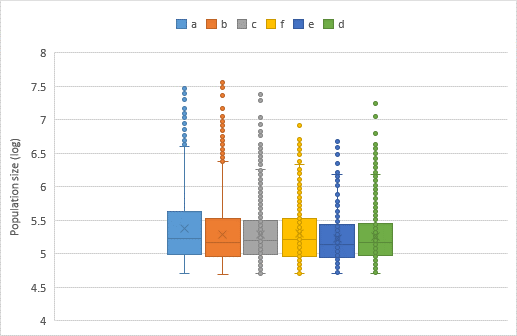


Figure S1 Frequency distribution of FUA by improving and worsening of LUE classes: improving classes (LUE_t2_ < LUE_t1_) a: was 0 < LUE_t1_ < 1 is 0 < LUE_t2_ < 1; b: was LUE_t1_> 1 is 0 < LUE_t2_ < 1; c: was LUE_t1_ > 1 is LUE_t2_ > 1; worsening classes (LUE_t2_ > LUE_t1_) d: was LUE_t1_ > 1 is LUE_t2_ > 1; e: was 0 < LUE_t1_ < 1 is LUE_t2_ > 1; f: was 0 < LUE_t1_ < 1 is 0 < LUE_t2_ < 1.

S3

Figure S2 Land Use Efficiency of FUA by proportion of their population in the Urban Centre

S4

Compactness of urban territories is typically computed by means of an indicator that relates the perimeter of the settlement with the area of the settlement. Among such indicators, the isoperimetric quotient proved to be effective for urban applications (Uhl et al., 2021). As per its definition, the isoperimetric quotient (*IPQ*) is calculated as:

$$IPQ=\frac{4\pi A}{p^{2}}$$

With A (km^2^) area of the urban settlement and p (km) its perimeter. For the purposes of this research work, the boundaries of urban settlements within FUAs are retrieved from the intersection of *Urban Clusters* with FUA boundaries. *Urban Clusters,* as defined through respective population density thresholds (Dijkstra et al., 2020), are obtained from the GHS-SMOD layer (Pesaresi et al., 2019) by merging class 1 (*Urban Centres*) with class 2 (*Urban Clusters*).


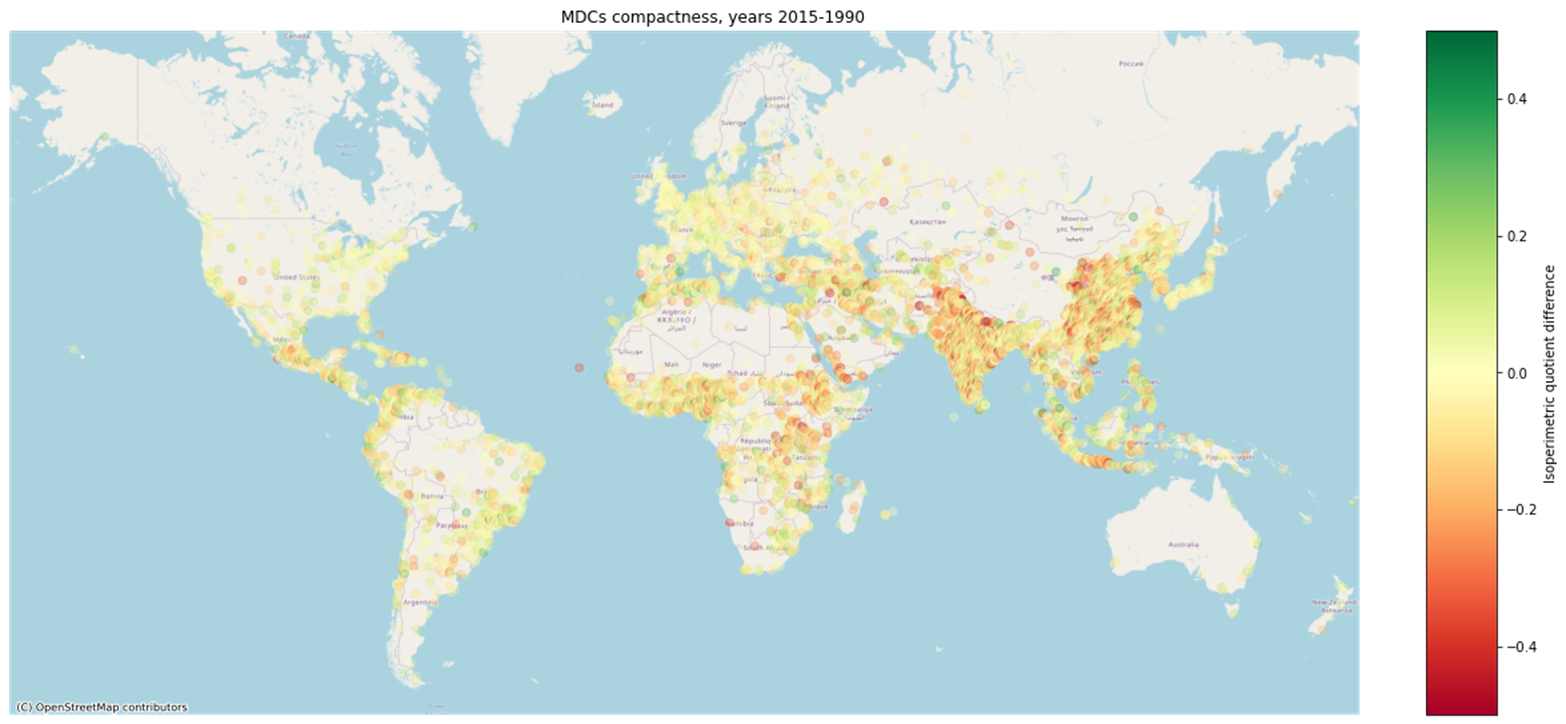


Figure S3 Compactness of Urban Clusters within FUAs, expressed as Isoperimetric quotient: the plotted variable is the difference between compactness in 1990 and in 2015. FUAs containing clusters with increased compactness shift to the green, those with decreased compactness shift to the red

**References**

Dijkstra, L., Florczyk, A. J., Freire, S., Kemper, T., Melchiorri, M., Pesaresi, M., & Schiavina, M. (2020). Applying the Degree of Urbanisation to the globe: A new harmonised definition reveals a different picture of global urbanisation. *Journal of Urban Economics*, 103312. https://doi.org/10.1016/j.jue.2020.103312

Pesaresi, M., Florczyk, A. J., Schiavina, M., Melchiorri, M., & Maffenini, L. (2019). *GHS settlement grid, updated and refined REGIO model 2014 in application to GHS-BUILT R2018A and GHS-POP R2019A, multitemporal (1975-1990-2000-2015), R2019A*. European Commission, Joint Research Centre (JRC). 10.2905/42E8BE89-54FF-464E-BE7B-BF9E64DA5218

Uhl, J. H., Connor, D. S., Leyk, S., & Braswell, A. E. (2021). A century of decoupling size and structure of urban spaces in the United States. *Communications Earth & Environment*, *2*(1), 20. https://doi.org/10.1038/s43247-020-00082-7
